# Application of different building representation techniques in HEC-RAS 2-D for urban flood modeling using the Toce River experimental case

**DOI:** 10.7717/peerj.11667

**Published:** 2021-07-02

**Authors:** Andam Mustafa, Michał Szydłowski

**Affiliations:** Faculty of Civil and Environmental Engineering, Gdansk University of Technology, Gdańsk, Poland

**Keywords:** Urban floods, Urban topography, HEC-RAS 2-D, Building representations, Numerical simulation, Hydrodynamic modeling

## Abstract

This paper presents the impact of the choice of building representation techniques and hydrodynamic models on urban flood simulations using HEC-RAS 2-D for the Toce River physical model. To this end, eight numerical models based on previous laboratory experiments were prepared to simulate unsteady urban flooding on each side of building units. Two simplified building layouts (aligned and staggered) were examined, where models were prepared for two different building representation techniques: Building Block (BB) and Building Resistance (BR). Water depth variation computations using the BR and BB techniques were compared to the laboratory measurements and previous studies in the literature. A statistical analysis was performed using both the Root Mean Square Error (RMSE) and the Pearson Product-Moment Correlation Coefficient (PPMCC) in order to evaluate the performance of the models. A sensitivity analysis showed that the proper mesh resolution and model parameter values were obtained. As far as the BR technique is concerned, it is well-suited for representing building units in numerical simulations using high Manning coefficients. Furthermore, this study confirms the importance of the BR technique, which should help researchers in using low-resolution Digital Elevation Models (DEMs) along with open-source programs. Moreover, the study aims to produce a deeper comprehension of numerical modeling and urban flooding.

## Introduction

Floods and flood modeling are a hot topic in the research field of hydrology and hydrological modeling. The Emergency Events Database EM-DAT of the Center for Research on the Epidemiology of Disasters (CRED) found that flooding caused the majority of disasters between 1994 and 2013, accounting for 43% of all reported events and affecting almost 2.5 billion people (CRED, 2015). Many researchers from different continents have studied the characteristics, effects and consequences of extreme rainfall events which happened under different hydrological circumstances (Arnbjerg-Nielsen, Leonardsen & Madsen, 2015; Ávila, Carvajal & Justino, 2015; Fu et al., 2013; Mustafa, Muhammed & Szydłowski, 2019; Rajeevan, Bhate & Jaswal, 2008; Ruin et al., 2014; Szpakowski & Szydłowski, 2018; Yilmaz, 2015). Moreover, rapid change in Land Use Land Cover (LULC) is considered to be a source of decreasing ground imperviousness, resulting in an increase in the amount of surface runoff. Recently, monitoring LULC changes using Remote Sensing (RS) has become an effective tool (Ali, Bohloul & Hosein, 2010; Apollonio et al., 2016; Mustafa & Szydłowski, 2020; Sanyal, Densmore & Carbonneau, 2014; Sharif et al., 2016; Shrestha, 2003; Zope, Eldho & Jothiprakash, 2016), and the Geospatial Information System (GIS) and hydrological modeling packages are a popular approach among researchers to delineate flood hazards and for flood mapping. Although, as human beings, we cannot control or stop such kinds of events, the frequency of occurrence and the level of damage could be effectively reduced through flood risk mapping, spatial planning and flood modeling using different techniques.

The choice of the hydrodynamic model is one of the crucial elements of flood modeling. In the literature, there are many studies dedicated to shallow-water equations (SWEs) for modeling flood inundation simulations in both Full Momentum and simplified models. The recommended one by the scholars is the Full Momentum model (Abderrezzak, Paquier & Mignot, 2009; Cunge, 2003). However, despite the availability of detailed, high-resolution topographic data, the lack of observed data prevents an adequate evaluation of the amount of data produced by any hydraulic model (Hunter et al., 2007). As a result, many research works applied the simplified so-called Diffusion Wave model for simulating urban flood events (Costabile, Costanzo & Macchione, 2017; Dottori & Todini, 2013; Fewtrell et al., 2011; Hunter et al., 2008; Prestininzi, 2008; Yu & Lane, 2006). Recently, the application of two-dimensional (2D) shallow-water equation (SWE) models is encouraged by the increasing availability of unique user-oriented computational codes (Pilotti et al., 2020). Teng et al. (2017) reviewed several well-known software/models that are capable of modeling flood inundation. Pender & Néelz (2010) compared the performance of some of the most common 2-D software.

The application of 2-D models is strongly required in built-up areas to reproduce the complex, multidirectional surface flow paths generated by urban configurations (Apel et al., 2009; Mignot, Paquier & Haider, 2006; Pina et al., 2016; Yu & Lane, 2011). In fact, flood propagation in urban areas is clearly two-dimensional, with peculiar characteristics that rely on complex interactions between the patterns of flow and streets/buildings, inducing multiple flow paths at intersections and flowing around or within buildings, etc. (Costabile et al., 2020a).

There are a number of 2-D numerical models and software packages with different capabilities and from different developers, some of which must be purchased and others which are open-source (Néelz & Pender, 2013). Based on the fact that 2-D hydrodynamic modeling is suitable for flood inundation in urban areas, in this study, the widespread modeling tool for hydraulic engineers, the open-source package of Hydrologic Engineering Center-River Analysis System (HEC-RAS) version 5.0.7, utilizing both Full Momentum and Diffusion Wave hydrodynamic models, has been applied. Before the 2016 update to version 5.0, HEC-RAS was one-dimensional, meaning that there was no direct modeling of the hydraulic effect of cross-section shape changes, bends and other two- and three-dimensional aspects of flow. In version 5.0, two-dimensional modeling of flow, as well as sediment transport modeling capabilities were introduced. In the literature, many studies have focused on utilizing HEC-RAS 2-D to generate flood inundation maps in urban areas (Abdelkarim et al., 2019; David & Schmalz, 2020; Haltas, Tayfur & Elci, 2016; Marko et al., 2018; Rangari, Umamahesh & Bhatt, 2019; Sharif et al., 2016; Surwase & Manjusree, 2019; Syafri, Hadi & Suprayogi, 2020; Szydłowski, 2019; Yalcin, 2020). Quirogaa et al. (2016) indicated the good performance of the inundation extent simulated by HEC-RAS 2-D when compared to that detected by a satellite image for the flood event February 2014 in the Bolivian Amazonia. In free-surface flow modeling, this program is very likely to become a norm, as is the well-known one-dimensional (1D) counterpart (Pilotti et al., 2020). Regardless of the different models, HEC-RAS codes can be governed by mesh representations, capabilities and input data requirements (Shustikova et al., 2019). Costabile et al. (2020b) investigated the performance and capabilities of the HEC-RAS 2-D model in basin-scale rainfall-runoff simulations. Moreover, they compared their results obtained using both the options (Full Momentum equations and Diffusion Wave equations) to the simulations obtained by using a 2-D Full Momentum model developed by the authors for research purposes. Ghimire, Sharma & Lamichhane (2020) evaluated one-dimensional (1-D) and two-dimensional HEC-RAS models to predict flood propagation time and inundation extent for a flood warning system. Shrestha et al. (2020) studied the suitability of MIKE 21 and HEC-RAS for 2-D floodplain modeling, and showed that overall, both software packages are perfectly capable as accurate flood management tools. Building on the existing literature, our study focuses on further deepening our knowledge and understanding of the potential and capabilities of the HEC-RAS 2-D model, to represent building units in an urban flood modeling simulation.

Built-up areas are characterized by a large number of buildings, streets, roads, and other manmade features. These are significant characteristics in urban environments, which should not be neglected during flood simulation. In order to obtain an accurate result for urban flood simulations, the mentioned characteristics should be simulated in an appropriate way. Soares-Frazão & Zech (2008) pointed out that flood propagation in urban areas is impacted by the building and street configurations. Using two physical experiments and a field study case, Jeong, Yoon & Cho (2012) numerically studied the effects of flood waves on urban areas due to a dam failure. Liu et al. (2018) used physical model experiments to reproduce the process of floodwater flowing around and through a house. Although declared before, Li et al. (2019) stated that buildings are one of the most important components in urban flood modeling, and their immovability to water flow is a critical factor in urban flooding. As well as the choice of the hydrodynamic model, the proper building representation technique is required for urban flood modeling. In the literature, up to now, four techniques have usually been applied to represent buildings in simulations, namely, Building-Hole (BH), Building-Block (BB), Building-Resistance (BR) and Building-Porosity (BP). In this paper, we investigate the application of the BB and BR techniques together with HEC-RAS 2-D for urban flood modeling.

In this study, the extent to which a 2-D hydrodynamic model (HEC-RAS) can be applied to simulate flash flood propagation in urban areas is investigated, using the different building representations. Previous experimental tests were used for the assessment of the model’s accuracy. We used data from the Toce River physical model, as obtained from Testa et al. (2007). This work attempts to fill some of the gaps highlighted above in the existing literature, feeding the debate described so far. Specifically, the aim of this study is to assess the potential and the capabilities of HEC-RAS 2-D to investigate the efficiency of different building representation techniques in terms of the accuracy of the representation of inundation processes within heterogeneous floodplains and the computational efficiency between the models with regard to different grid resolutions and roughness coefficients. In addition, we used the results presented by Szydłowski (2005) to verify the different building representation techniques in numerical simulations of urban flooding. The main objectives of this study were:

- To apply two (BB and BR) building representation techniques available in HEC-RAS 2-D and verify their applicability for urban flood modeling.
- To compare the urban flood simulation results obtained from different hydrodynamic models (2-D Saint-Venant and 2-D Diffusion Wave).
- To assess the impact of building layout on the quality of numerical results.
- To investigate the impact of mesh resolution on the outcomes.

This study investigates and verifies the applicability of a high Manning roughness coefficient, the Building-Resistance (BR) method, for a built-up area flood simulation in HEC-RAS 2-D. Previously, similar techniques were tested by Beretta et al. (2018). However, the authors focused only on steady flow in their work. In our research, we model and investigate unsteady flood wave propagation. Moreover, our conclusions regarding the Diffusion Wave model, applied for urban flood modeling, are different from those presented before. This study should help researchers that use low-resolution DEMs for urban flood modeling to implement this technique along with an open-source program such as HEC-RAS 2-D. Moreover, the research aims to provide a deeper comprehension of numerical modeling and urban flooding.

## Materials & Methods

### Toce river physical model

**Figure 1:**
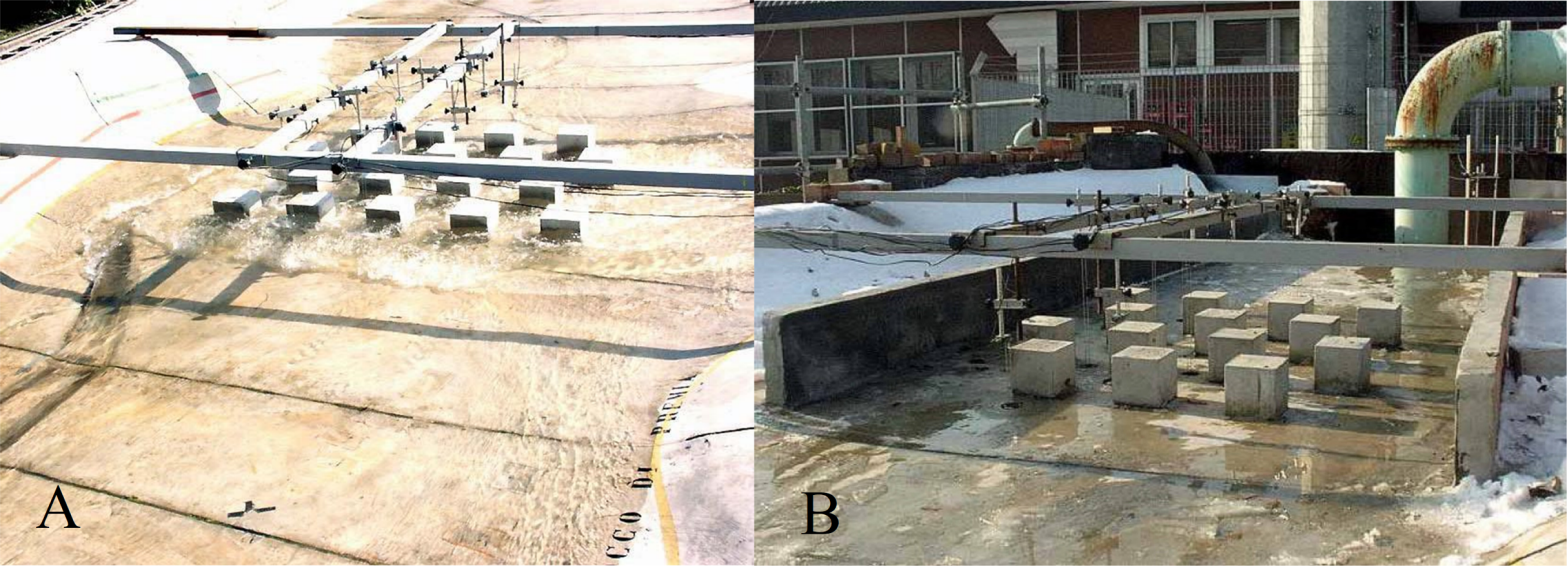
(A) The upper part of the original model where the buildings are laid out in the staggered configuration. (B) Bathymetric set up by the placement of two masonry walls, where the model city has been located (staggered configuration).

**Figure 2:**
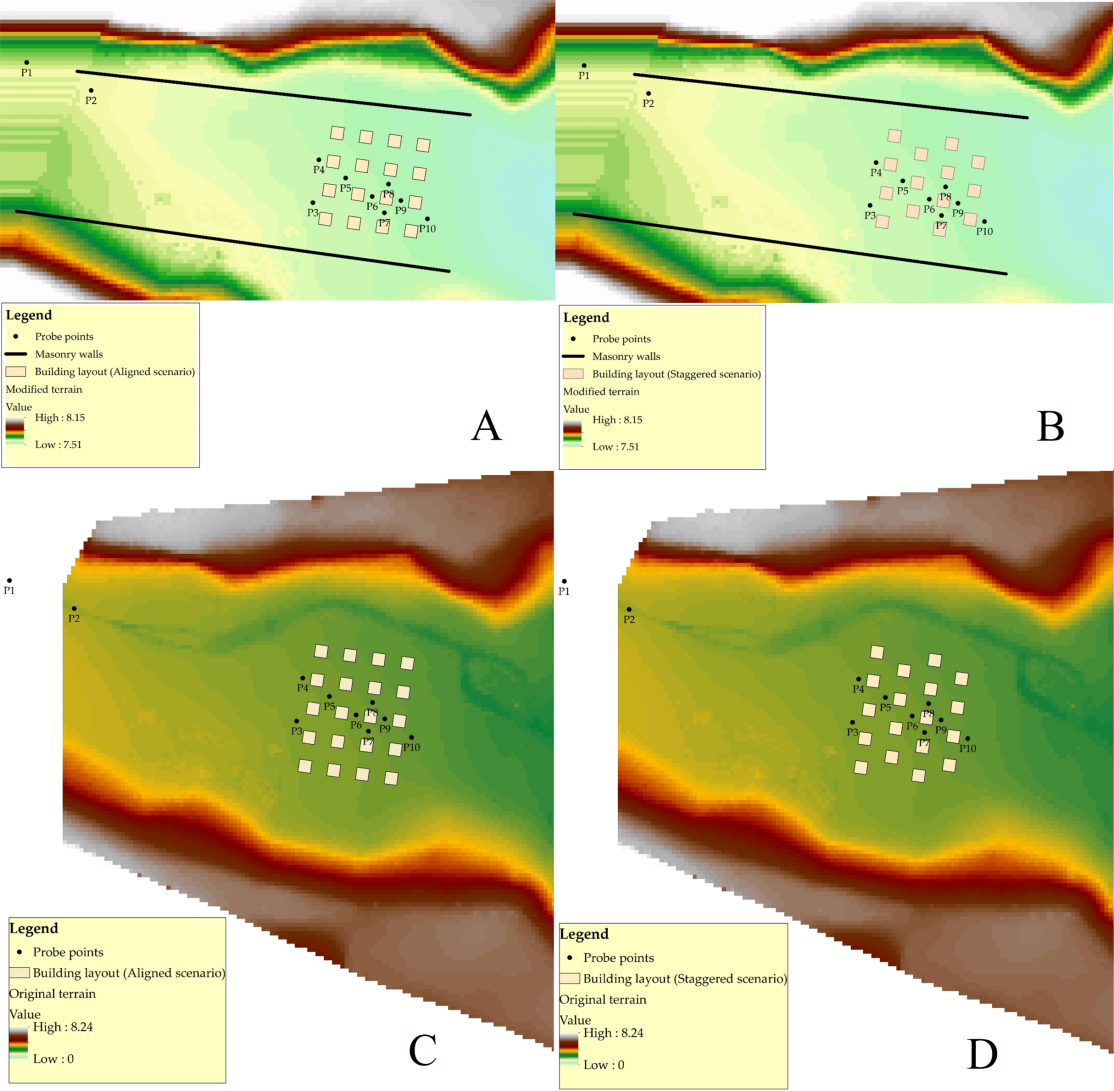
(A) The aligned layout of buildings in the modified DEM. (B) The staggered layout of buildings in the modified DEM. (C) The aligned layout of buildings in the original DEM. (D) The staggered layout of buildings in the original DEM.

The model of the Toce River was built at the hydraulic laboratory of ENEL-CESI in Italy, and was used to investigate the dam-break flow in the natural valley (Testa et al., 2007). The physical model was created in concrete at a scale of (1:100) for 5 km of the Toce River (Fig. 1A). The model dimensions were 50 m long and 11 m wide in quite geographical detail with water depth gauges at appointed locations. Testa et al. (2007) presented the same Toce River model with only the part containing urban buildings. In order to simplify the flow structure, the urban zone was isolated from the valley borders by two masonry walls positioned parallel to the model’s main axis (the distance between two walls varies from 185 cm at the upstream to 190 cm at the downstream), and it included urban buildings that were represented by 15 × 15 cm concrete cubes (Fig. 1B). In this study, both original and modified (with placing two parallel walls) setups were studied. The physical model had a digital terrain model of 5 cm. However, during modeling in this study, the digital terrain model was reproduced at a higher resolution of 1 cm, as well as producing a modified digital terrain model on the base of the wall and urban area. The water depth data were recorded at 10 locations (Figs. 1A and 1B and Figs. 2A–2D) using electrical conductivity gauges. During the experiment, the measurement instruments recorded the water level at 0.2 s intervals, providing enough high temporal resolution for the validation of the model. The gauges were suspended above the model so that they did not interpose with the flood wave.

Generally, 10 electrical gauges were used, two of them, P1 and P2, were located at the entrance point of the flood discharge, and the rest, P3–P10, were placed beside the concrete blocks in order to record water depth variations. The experiments were done in two different topographical forms (original and modified). In the modified DEM, only a 7-m-long region situated at the upstream end of the physical model was simulated. Additionally, two building layouts in the model of the urban area were assessed:

Aligned layout, including 16 buildings located in rows with the radial direction approximately parallel to the main axis of the valley for the modified DEM, but for the original DEM, including 20 buildings (Figs. 2A and 2C).

Staggered layout, including only 14 buildings located in a checkerboard layout for the modified DEM, while for the original DEM, including 18 buildings (Figs. 2B and 2D).

**Figure 3:**
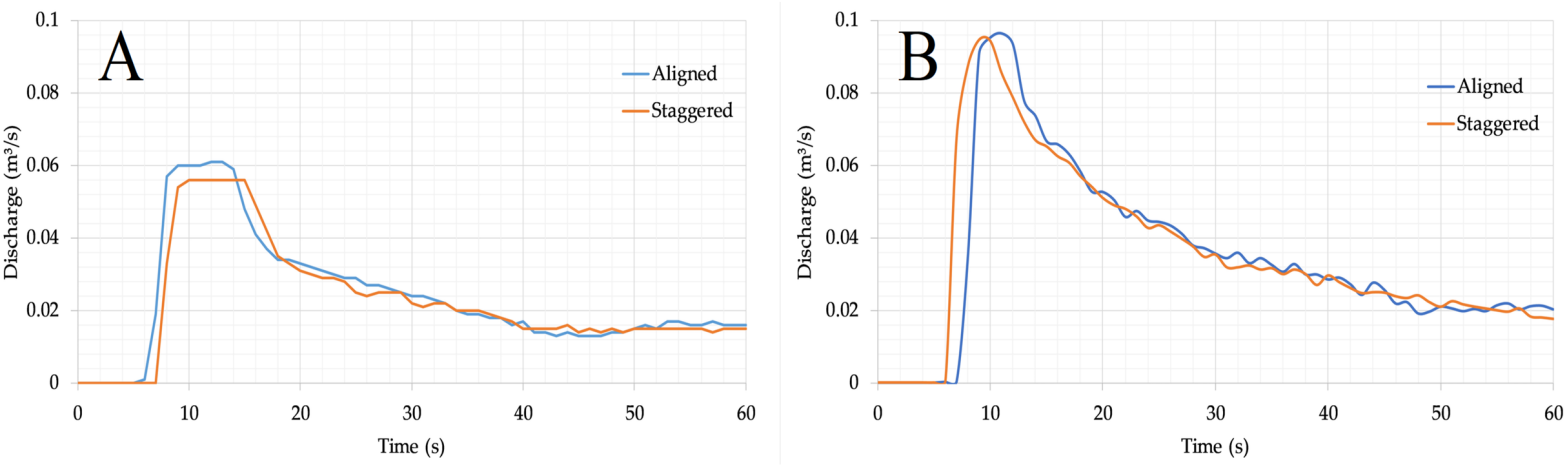
(A) Inflow discharge hydrograph for the modified DEM. (B) Inflow discharge hydrograph for the original DEM.

The flood hydrographs recorded as the inflow discharge for both the modified and original DEMs, and in the two cases of aligned and staggered building layouts, are shown in Figs. 3A and 3B. These two flow discharge hydrographs, for both layouts respectively, are quite similar, even though some differences can be noticed regarding the flow peak. Measurements of the depth variation at points P1 and P2 were also used to define the boundary condition at the inflow section. During the experiments, the flow through the inlet section was subcritical. At the downstream end of the model, the outflow is sub- or supercritical depending on the actual flow conditions.

### HEC-RAS model

HEC-RAS is an open-source software that models the hydraulics of water flow through natural rivers and other channels, developed by the U.S. Army Corps of Engineers (USACE). This software allows the user to perform one-dimensional steady and unsteady flow modeling, 2-D unsteady flow modeling, as well as combined 1-D and 2-D unsteady flow routing, sediment transport/mobile bed computations, and water temperature/water quality modeling (Brunner, 2016a). The HEC-RAS 2-D unsteady flow equation solver uses an implicit finite volume algorithm. The implicit solution algorithm enables a larger computational time step than the explicit method. The program was designed for the application of unstructured computational mesh, but can also handle structured mesh. HEC-RAS 2-D presents two types of computational approaches in order to compute the flow field in the defined 2-D mesh: Full Momentum (Saint-Venant) equations and the Diffusion Wave model. The 2-D Full Momentum equations solver takes into account the turbulence and Coriolis effects in the flow field and thus needs greater computational power and more time to perform the simulations. Furthermore, a very fine mesh is required to overcome rapid changes in the direction of flow within the 2-D flow field. The numerical mesh prepared for the simulation in this study was at the mesh sizes of 1, 2 and 5 cm, and composed of 92,403, 23,014 and 3,641 computational cells, respectively. The Diffusion Wave model requires less time and promises higher model stability but at the expense of the precision of the results (Quirogaa et al., 2016). Artichowicz & Gąsiorowski (2019) presented a study that deals with increasing the computational efficiency in modeling floodplain inundation by using a two-dimensional Diffusion Wave equation. In the present study, both model options (the Full Momentum equations and the Diffusion Wave) were considered for HEC-RAS 2-D. The solved 2-D Saint Venant equations in their non-conservative forms are as follows:

(1) \displaystyle{{\partial H} \over {\partial t}} + \displaystyle{{\partial \left( {hu} \right)} \over {\partial x}} + \displaystyle{{\partial \left( {hv} \right)} \over {\partial y}} + q = 0

(2) \displaystyle{{\partial u} \over {\partial t}} + u\displaystyle{{\partial u} \over {\partial x}} + v\displaystyle{{\partial u} \over {\partial y}} = - {\rm g}\displaystyle{{\partial H} \over {\partial x}} + {v_t}\left( {\displaystyle{{{\partial ^2}u} \over {\partial {x^2}}} + \displaystyle{{{\partial ^2}u} \over {\partial {y^2}}}} \right) - {c_f}u + fv

(3) \displaystyle{{\partial v} \over {\partial t}} + u\displaystyle{{\partial v} \over {\partial x}} + v\displaystyle{{\partial v} \over {\partial y}} = - {\rm g}\displaystyle{{\partial H} \over {\partial y}} + {v_t}\left( {\displaystyle{{{\partial ^2}v} \over {\partial {x^2}}} + \displaystyle{{{\partial ^2}v} \over {\partial {y^2}}}} \right) - {c_f}v + fu

where *t* is time, *u* and *v* are considered as the velocity horizontal components in *x* and *y* direction, respectively, and *q* is a source/sink flux term. }{}H is water surface elevation, }{}h is water depth, *g* is gravitational acceleration, }{}{v_t} is the coefficient of horizontal eddy viscosity, }{}{c_f} is the coefficient of bottom friction, and }{}f is the Coriolis parameter. A full explanation of the equations can be found in the HEC-RAS version 5.0 hydraulic reference manual (Brunner, 2016b).

### Building representation techniques

Up to now, in the literature, four building representation techniques have usually been used to model built-up area flooding in hydrodynamic numerical models (Aronica & Lanza, 2005; Brown, Spencer & Moeller, 2007; Gallien, Schubert & Sanders, 2011; Guinot, 2012; Hunter et al., 2008; Li et al., 2019; Schubert & Sanders, 2012; Schubert et al., 2008; Szydłowski, 2005), as follows:

### Building-block technique

In this method, the ground elevation of the building units should be increased by modifying the distributed ground elevation data, by way of configuring the buildings to a real height or a sufficiently large artificially high elevation value to ensure that no water flows over the buildings. Herein, the whole simulated flow area should be meshed as a unified grid, without missing grids, so the water flows around the buildings. However, this method requires grid refinement around the buildings in order to precisely represent building profiles. This technique can be applied in HEC-RAS 2-D using sufficiently fine-structured or unstructured 2-D numerical mesh. It is absolutely essential to have a detailed and accurate DEM in order to create a detailed and accurate hydrodynamic model for built-up areas. The precision of the DEM can be a limiting factor regarding the quality of the hydraulic model that the user can create (Brunner, 2016a).

### Building-resistance technique

In this method, the modeler must allocate a different Manning coefficient to each grid according to the requirements. When the user inputs a high Manning coefficient, as a result, a low water flow velocity will appear. In such a method, a high Manning *n* value is set to the simulated building areas to artificially increase the resistance of the buildings against the water flow. While for the other simulated areas, a reasonably low value is set which should represent the real land cover. Thus, the water flows slowly over the building units but the flow regime behaves as if there is an obstacle, which is because of the high resistance coefficient assigned to the building units. This technique is useful when obtaining a high-resolution DEM is difficult or expensive. The BR technique is applicable in HEC-RAS 2-D. The user can create their own 2-D area (user-defined polygons), known as Manning coefficient *n* value regions, in which the Manning coefficient *n* value from the LULC data set can be overridden. Here, the user must have a LULC map in order to utilize the spatially varying Manning *n* value within the delineated 2-D flow area, and also to use the capability of specifying the user-defined Manning *n* region.

### Building-hole technique

In this case, the buildings are treated as holes in a numerical mesh representing the flow area. The mesh holes are positioned with a building layout where free-slip wall conditions create a blockage effect, which means that the water flow would neither overflow nor permeate over the buildings. The model simulates the water flow for all the grids except the buildings which are represented by holes. Nevertheless, when building geometries are complex, the method can produce undesirable mesh refinements that significantly degrade the model efficiency (Mason et al., 2007; Schubert et al., 2008; Tsubaki & Fujita, 2010). In HEC-RAS 2-D, there is no possibility to deactivate a cell and/or cells or include holes inside the 2D flow area, which is why this technique cannot be applied.

### Building-porosity technique

This technique, adopted from the porous media theory, leads to a modification of Saint-Venant equations (Guinot, 2012). Porosity can be explained in several ways, for instance, as a volume average portion of pore space in a permeable medium or as an areal average portion of pore space, as in a slice through a permeable medium (Bear, 1988). Both volumetric and areal porosity can be expected to change spatially in the case of a nonhomogeneous permeable medium, and areal porosity can also vary with the orientation of the surface over which the areal average is taken, and can consequently show anisotropy. If an urban surface area full of solid features is taken as a permeable medium, then the pore space reflects the gaps between the solid features, the volumetric porosity represents the portion of the land surface able to store water, and the areal porosity reflects the fraction of space convenient for directionally dependent flood conveyance (Kim et al., 2015). However, this technique is not applicable in HEC-RAS 2-D, because only the standard Full Momentum (Saint Venant equations) and Diffusion Wave models are applied in this software.

## Results

Flood simulation can be performed using one of many approaches, which differ in process representations and numerical models. In this study, eight test cases were prepared. The test cases were created for the original Toce River physical model and a modified one, for two kinds of building configurations (aligned and staggered), with two types of building representations (BB and BR) for each model, available in HEC-RAS 2-D. In the numerical simulations, the same hydraulic conditions as observed during the physical modeling were investigated. The issue of physical scaling was not analyzed in the modeling process. The Toce River physical model was considered as a full-size hydraulic system. The concept of the study was firstly to identify the models and techniques and secondly to verify them. To this end, the modified DEM was used in the identification stage, and the original DEM was used in the verification stage. An unsteady flow, allowing the analysis of flood wave propagation, was considered for all the tests. Table 1 presents the naming order of test cases.

**Table 1:** The naming of test cases in the study.

Test case	Building layout	DEM	Building representation
1a-BB	Aligned	Modified	Building Block (BB)
1b-BB	Staggered	Modified	Building Block (BB)
1a-BR	Aligned	Modified	Building Resistance (BR)
1b-BR	Staggered	Modified	Building Resistance (BR)
2a-BB	Aligned	Original	Building Block (BB)
2b-BB	Staggered	Original	Building Block (BB)
2a-BR	Aligned	Original	Building Resistance (BR)
2b-BR	Staggered	Original	Building Resistance (BR)

The 2-D flow area in HEC-RAS 2-D is delineated to generate a 2-D numerical mesh by outlining a polygon within the boundary of the underlying DEM. To precisely simulate the terrain and profile of the buildings, 1, 2 and 5 cm mesh resolutions were tested and compared, using a 0.02 s time step. Mesh resolution is considered as one of the most important parameters in numerical models (Horritt & Bates, 2001; Yu & Lane, 2006). The model simulation time step, together with the mesh size, determines the simulation run time and accuracy in mapping the outputs (Rangari, Umamahesh & Bhatt, 2019). In the BB method, the elevation of the building blocks was increased to imitate reality. In the BR method, the Manning coefficient was set to 0.1, 1 and 10 m^−1/3^⋅s for the building grids, and 0.0162 m^−1/3^⋅s for the other grids; the last value of the Manning coefficient is suggested by the experimental team at ENEL-CESI for the concrete bed (Testa et al., 2007).

### Analysis of different mesh resolutions in the BB technique

The Building Block (BB) technique as a representative of building units in hydrodynamic numerical modeling, is considered primarily as the most realistic method. The main factor that affects the outcome of hydrodynamic model simulations is geometric information. Due to the fact that the elevation of the built-up area is increased or high enough in the simulation model so that neither water storage nor water flows over the buildings in the flow field. In order to analyze this method in detail, the impact of the mesh resolution on the numerical modeling results is evaluated, which would give a better understanding of numerical modeling in composite urban scenarios. In the test cases (numbers 1a-BB and 1b-BB), the numerical mesh prepared for the simulation at the mesh sizes of 1, 2 and 5 cm, and composed of 92,403, 23,014 and 3,641 computational cells, respectively, was to represent the modified DEM (Figs. 2A and 2B).

**Figure 4:**
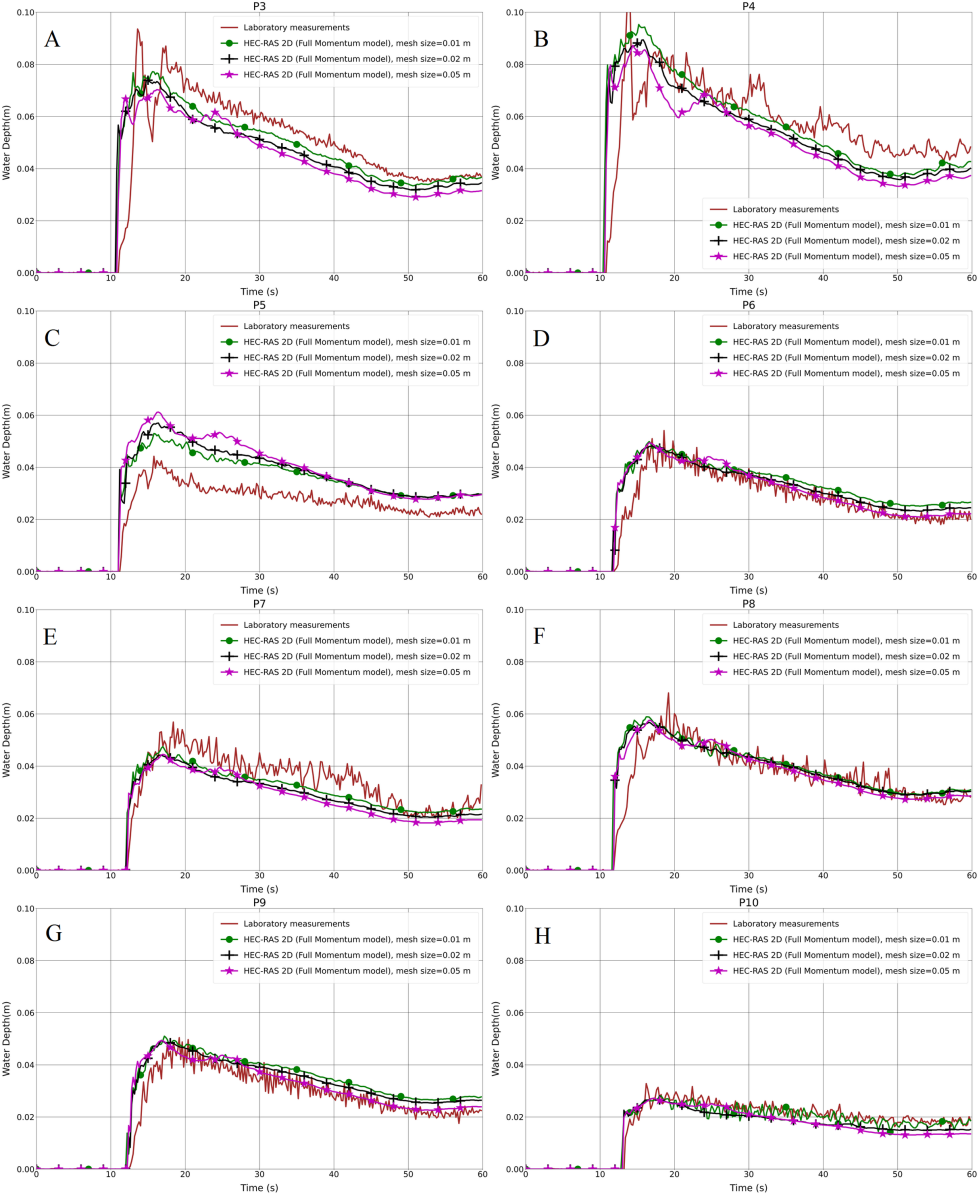
(A–H) Analysis of the numerical simulation using different mesh resolutions for the modified DEM with the aligned building layout (1a-BB).

**Figure 5:**
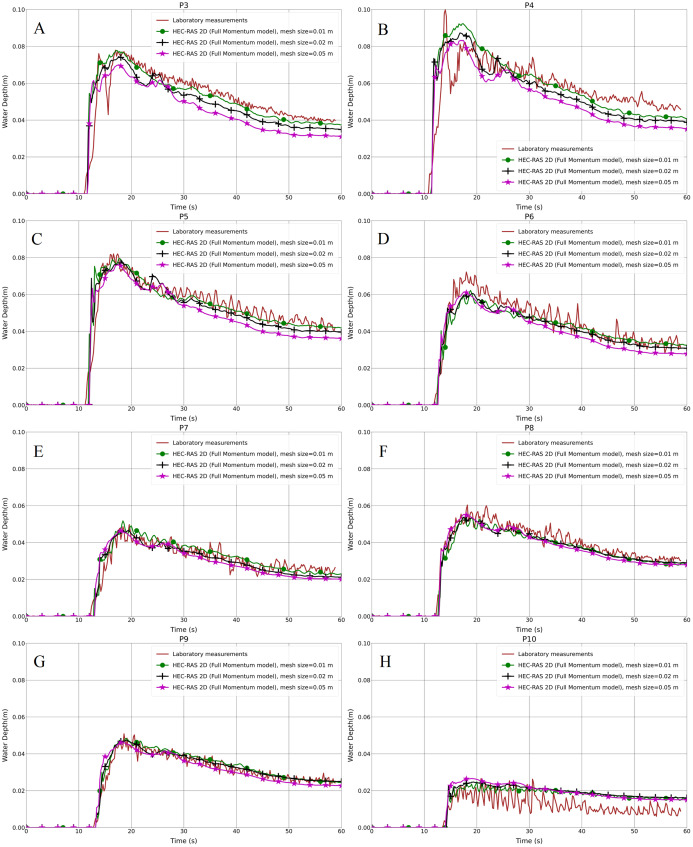
(A–H) Analysis of the numerical simulation using different mesh resolutions for the modified DEM with the staggered building layout (1b-BB).

The resulting water depth variation in the hydrodynamic model (Full Momentum) and the laboratory measurements for both the aligned and staggered scenarios are shown in Figs. 4A–4H and 5A–5H, respectively. As can be seen in the graphs, the front of the flood waves hits and passes the first building row after almost 11 s of simulation. Generally, the predicted results of the water depth for the 5 cm mesh size model were lower than the 1 and 2 cm grid models in both the aligned and especially the staggered case, with the peak water depth values illustrating the same trend. Further analysis showed that the water depth at P5 in the aligned case with all grid resolutions is higher than the laboratory measurements (Fig. 4C). The reason for this disagreement may be because of an error in measurement which could not be verified at this stage, or it may be the outcome of the specific location of point P5 near to which water swelling is noticed. The depth results of the 1 cm mesh resolution are marginally higher than the 2 cm mesh resolution. Furthermore, as can be noticed in the 1 cm grid resolution results, there is obvious data oscillation, especially at P9 and P10, which could be considered as the efficiency and accuracy of the model compared to the 2 and 5 cm grid models (Figs. 4G–4H and 5G–5H).

**Figure 6:**
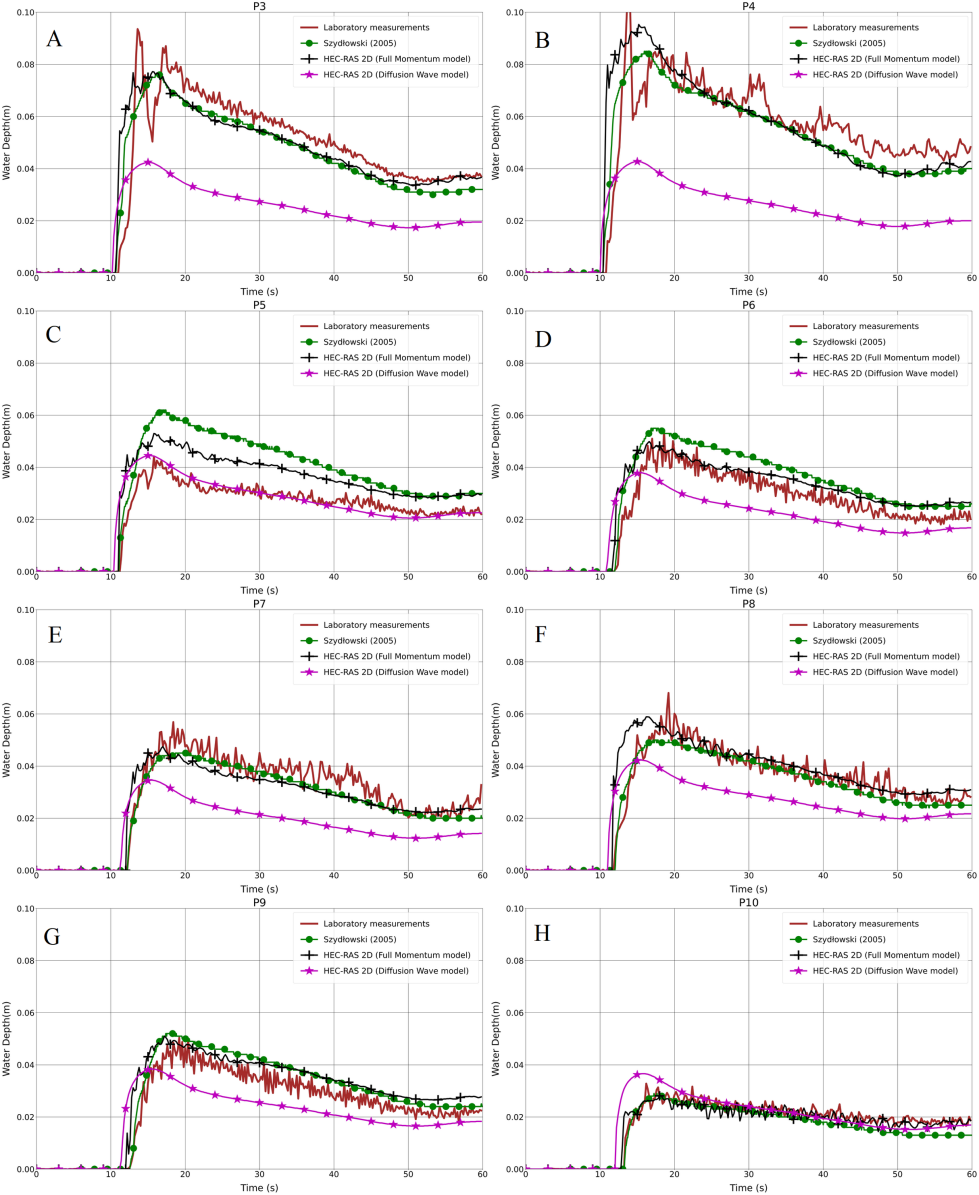
(A–H) Laboratory measurements compared to both the Full Momentum model and the Diffusion Wave model, as well as to the solution by Szydłowski (2005), for the modified DEM with the aligned building layout (1a-BB).

**Figure 7:**
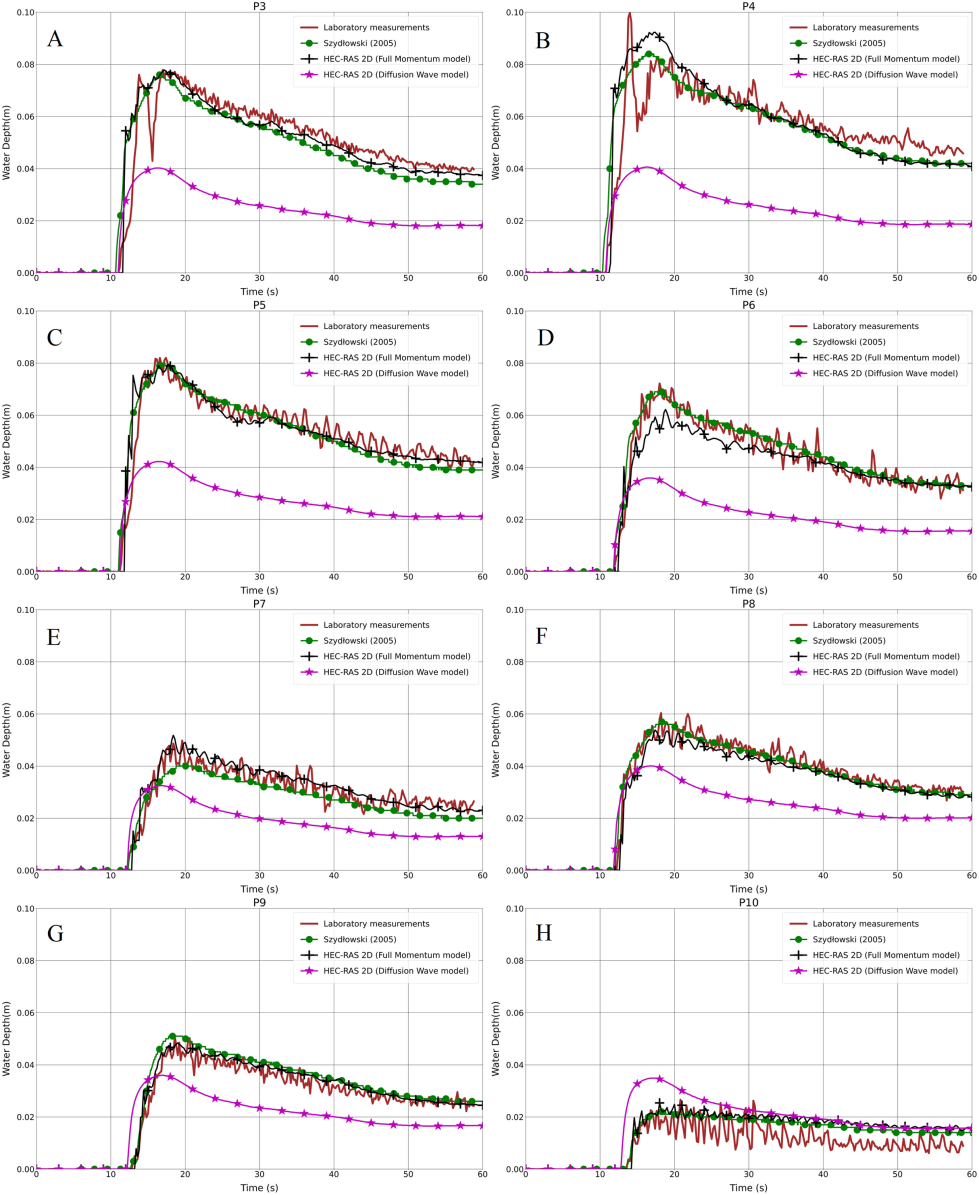
(A–H) Laboratory measurements compared to both the Full Momentum model and the Diffusion Wave model, as well as to the solution by Szydłowski (2005), for the modified DEM with the staggered building layout (1b-BB).

In the second step for the same test cases (1a-BB and 1b-BB), with the 1 cm mesh size and the BB representation technique, we compared the results from the Full Momentum and Diffusion Wave models with the laboratory measurements. In Figs. 6A–6H and 7A–7H, it is clearly noticeable that the water depth in the Diffusion Wave model at all probe points was underestimated, except P10, which is the point where the curve fits the laboratory measurement curve. The agreement and discrepancies of the water level at P5 with the laboratory measurements and numerical solutions are explained earlier in this section (Fig. 4C). Generally, the reason for the disagreement of the Diffusion Wave model is that it is a simplified model and could not accurately calculate the water swelling and dynamics due to no representation of inertia forces in the dynamic equations. The simulation solving the 2-D Diffusion Wave equations was faster, which takes about 45% of the time required to simulate the same model using the Full Momentum model. But the results were underestimated, compared to the observed laboratory values.

Regarding the statistical presentation of the outcomes of our work, we used both the Root Mean Square Error (RMSE) and the Pearson Product-Moment Correlation Coefficient (PPMCC) in order to evaluate the performance of the models, for instance, the difference between 1, 2 and 5 cm mesh resolutions. Overall, the RMSE of the 1 cm mesh resolution is lower than the 2 cm one, and the 5 cm mesh resolution is the highest in both the aligned and staggered building layouts (Table 2). The PPMCC of the 1 cm mesh size is the highest compared to the 2 and 5 cm mesh resolutions. However, the difference is very small. Yu & Lane (2006) stated that mesh resolution affects model performance; whenever the mesh is finer, the results will be better. But also decreasing the mesh resolution further causes an output of data oscillation, affecting the accuracy of the results. Therefore, it is better to find the proper mesh resolution according to the building layout, due to the fact that, generally, 1 cm mesh resolution outputs are the closest ones to laboratory results compared to 2 cm and 5 cm resolutions. Moreover, we verified that a simulation with the grid size smaller than 1 cm will not change the results remarkably. In contrast, it takes a longer time and causes instability in the simulation. Based on Figs. 4A–4H and 5A–5H and Tables 2 and 3, we can conclude that the 1 cm mesh resolution is the proper grid size for the Toce River physical model.

**Table 2:** Calculated RMSE for both building layouts and different grid sizes (cm) (1a-BB and 1b-BB).

Building layout	Grid size (cm)	P3	P4	P5	P6	P7	P8	P9	P10
Aligned (1a)	1	1.00	1.32	0.92	0.60	0.57	0.72	0.63	0.29
	2	1.13	1.26	1.09	0.53	0.66	0.64	0.56	0.37
	5	1.19	1.38	1.27	0.52	0.73	0.62	0.55	0.40
Staggered (1b)	1	0.83	1.09	0.78	0.68	0.48	0.54	0.43	0.65
	2	0.88	1.08	0.79	0.65	0.47	0.54	0.42	0.71
	5	1.09	1.18	0.91	0.73	0.53	0.54	0.47	0.71

**Table 3:** Calculated PPMCC for both building layouts and different grid sizes (cm) (1a-BB and 1b-BB).

Building layout	Grid size (cm)	P3	P4	P5	P6	P7	P8	P9	P10
Aligned (1a)	1	0.92	0.88	0.98	0.96	0.96	0.93	0.97	0.96
	2	0.90	0.90	0.97	0.96	0.96	0.94	0.97	0.96
	5	0.91	0.88	0.96	0.95	0.95	0.94	0.95	0.95
Staggered (1b)	1	0.96	0.94	0.98	0.98	0.98	0.99	0.99	0.87
	2	0.96	0.94	0.98	0.99	0.97	0.99	0.99	0.88
	5	0.95	0.93	0.97	0.98	0.96	0.99	0.97	0.90

### Analysis of different Manning coefficients in the BR technique

The Manning coefficient is a coefficient describing the roughness or friction of a surface in the field of flow, which estimates the average flow velocity. Because it is an empirical coefficient, *n* values are often selected from tables, but can also be calculated from field measurements. In many flow cases, the value of the Manning roughness coefficient has a significant effect on the computational results. In the BR technique, three different Manning coefficient values (such as 0.1, 1 and 10 m^−1/3^⋅s) are assigned to all the mesh regions that represent the building blocks (which here are known as user-defined polygons) to examine the resistance against the flow. Only one “high” Manning value is used in any given simulation run. In the test case (numbers 1a-BR and 1b-BR), the model was prepared for the simulation with the mesh size of 1 cm, composed of 92,403 computational cells.

**Figure 8:**
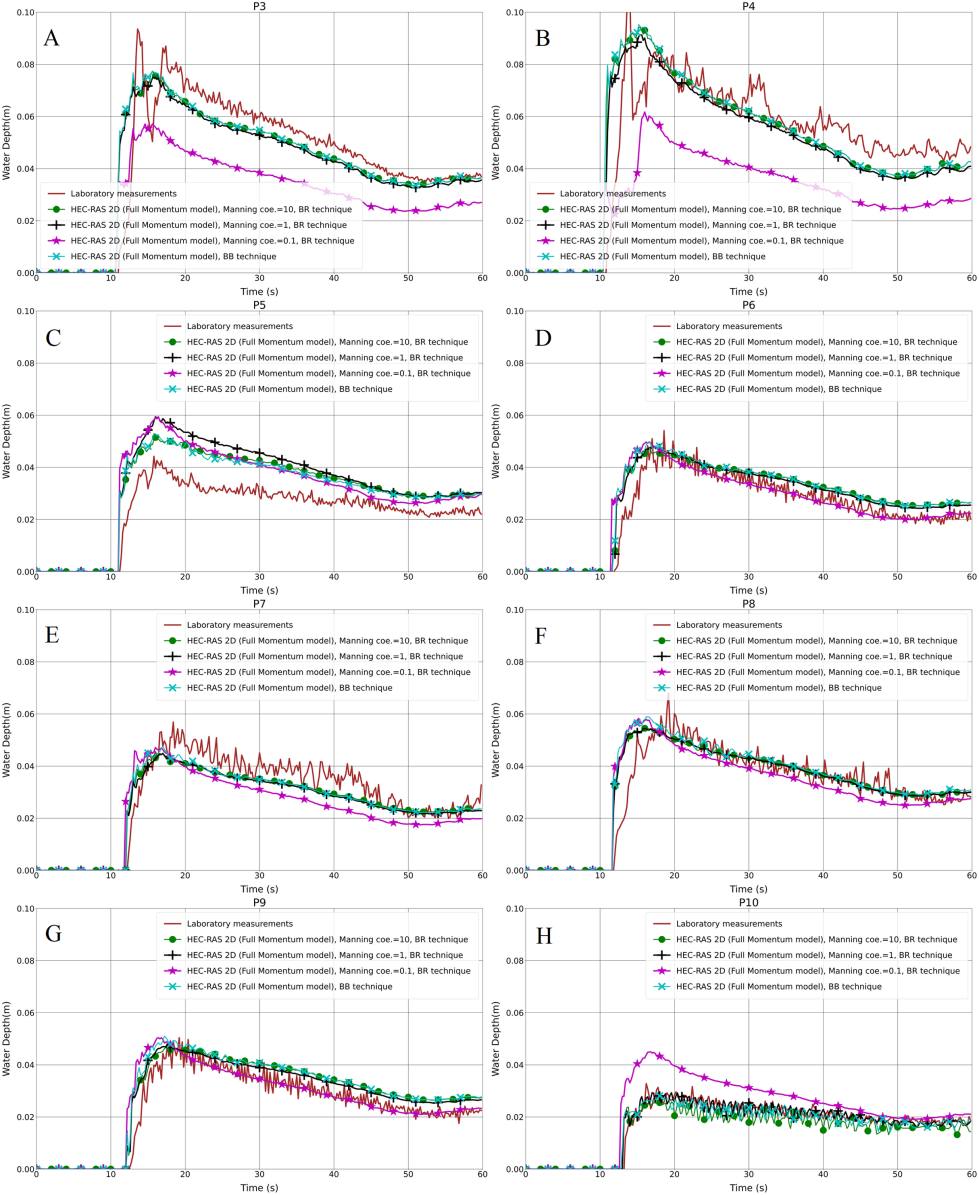
(A–H) Analysis of the numerical simulation using different Manning values for the modified DEM with the aligned building layout (1a-BR).

**Figure 9:**
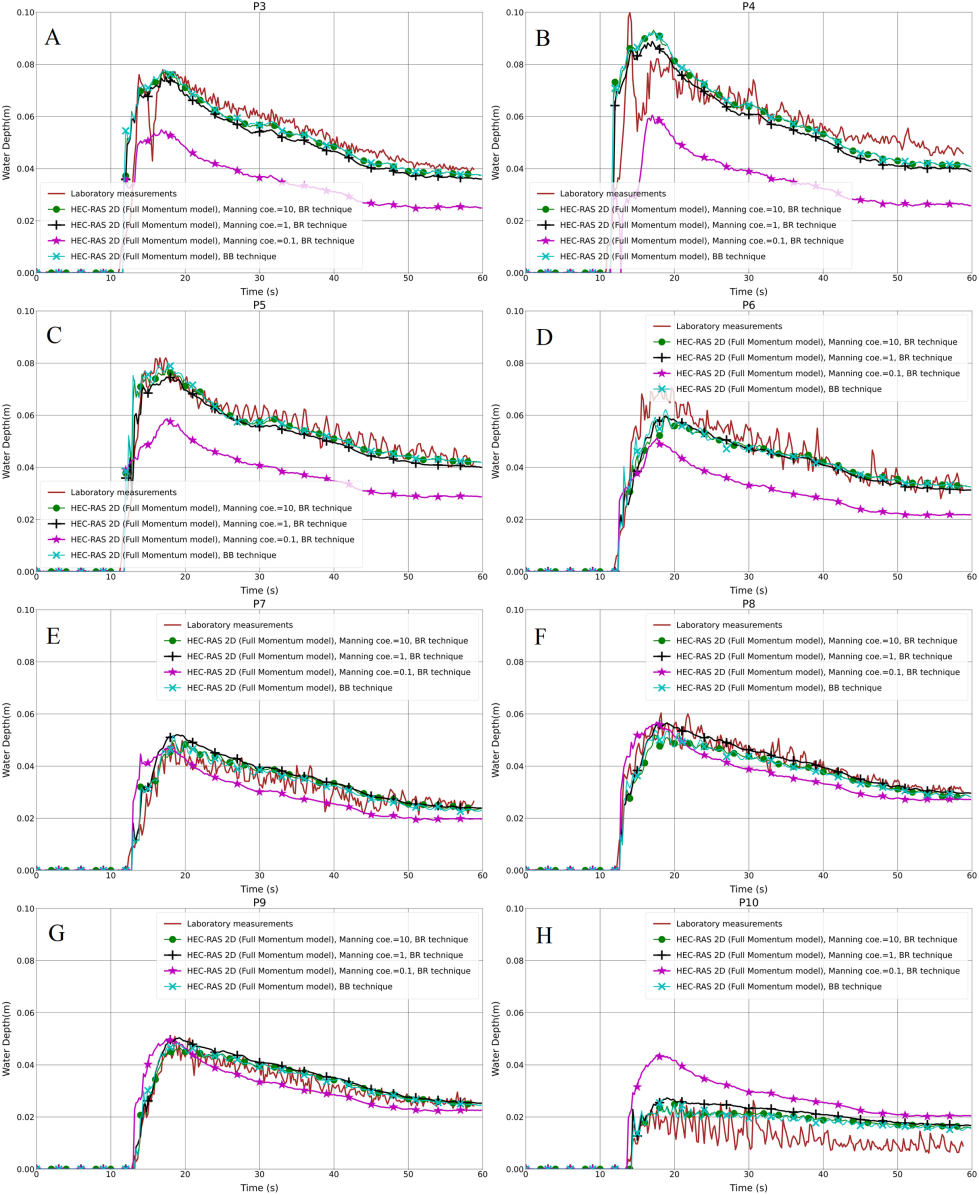
(A–H) Analysis of the numerical simulation using different Manning values for the modified DEM with the staggered building layout (1b-BR).

The resulting water depth in the hydrodynamic models and the laboratory measurements for both the aligned and staggered scenarios are shown in Figs. 8A–8H and 9A–9H, respectively. The propagation time from the inflow to the built-up area was the same time as in the BB method; the front of the flood wave hits and passes the first building row after almost 11 s of simulation. In the aligned configuration, at the probe points P3, P4 and P7, the outcome of the water depth in the simulation with the Manning coefficient, equal to 0.1 m^1/3^⋅s, is underestimated compared to the laboratory measurements; P5 and P10 are overestimated, and the rest of the points P6, P8 and P9, are in quite good accordance with the experimental results (Figs. 8A–8H). In the staggered layout (Figs. 9A–9H), the water depth outcome at P10 is overestimated, and at P9 the advantage of the cyan (line with x marker) and black (line with plus sign marker) lines over the magenta (line with star marker) one is very small; the water depth outputs at the remaining of probe points are underestimated. Simultaneously, the black lines with plus sign markers and the green lines with filled circles in the graphs, which are the outputs of the simulations with the Manning coefficient equal to 1 and 10 m^−1/3^⋅s, respectively, fit well the laboratory measurements. This indicates that the building blocks are resistant to the water flow in the flow field by their physical nature. Moreover, different Manning coefficient simulation models are compared to the BB technique in all the graphs. The plots showed a very good representation of BB in the case of the simulation model with the Manning coefficient equal to 10 m^−1/3^⋅s. There is a small difference between the simulation models with the Manning coefficients equal to 1 and 10 m^−1/3^⋅s, but this could be neglected.

Turning to the statistical presentation of the results of this part, the RMSE is considered as the standard way to measure the error of a model in predicting quantitative data, thus we evaluated the prepared models for usefulness and accuracy. The RMSE of the Manning coefficient 0.1 is higher than those of 1 and 10 m^−1/3^⋅s in both the aligned and staggered building layouts (Table 4). The difference between the RMSEs of the Manning coefficients 1 and 10 m^−1/3^⋅s is very small, while there is a noticeable difference between them and the model with the Manning coefficient 0.1 m^−1/3^⋅s. The PPMCC of the Manning coefficient 0.1 is the lowest compared to those of 1 and 10 m^−1/3^⋅s. However, the difference is very small (Table 5).

**Table 4:** Calculated RMSE for both building layouts and different Manning values (cm) (1a-BR and 1b-BR).

Building layout	Manning coefficient value	P3	P4	P5	P6	P7	P8	P9	P10
Aligned (1a)	0.1	1.76	2.32	1.06	0.58	0.87	0.77	0.58	0.79
	1	1.02	1.21	1.21	0.52	0.57	0.62	0.51	0.25
	10	0.97	1.24	0.96	0.55	0.55	0.62	0.56	0.36
Staggered (1b)	0.1	1.84	2.29	1.66	1.42	0.64	0.58	0.51	1.42
	1	0.68	0.88	0.46	0.53	0.46	0.29	0.32	0.80
	10	0.62	0.91	0.48	0.95	0.38	0.40	0.25	0.66

**Table 5:** Calculated PPMCC for both building layouts and different Manning values (cm) (1a-BR and 1b-BR).

Building layout	Manning coefficient value	P3	P4	P5	P6	P7	P8	P9	P10
Aligned (1a)	0.1	0.93	0.92	0.95	0.92	0.91	0.91	0.93	0.92
	1	0.92	0.90	0.97	0.97	0.97	0.94	0.97	0.97
	10	0.92	0.89	0.98	0.96	0.96	0.94	0.97	0.96
Staggered (1b)	0.1	0.96	0.93	0.97	0.97	0.91	0.97	0.95	0.90
	1	0.97	0.95	0.99	0.98	0.98	0.99	0.99	0.87
	10	0.97	0.94	0.98	0.94	0.98	0.99	0.99	0.86

### Verification of the modeling techniques

The concept of the study was to identify models and techniques for the modified geometry of the Toce River physical model (Figs. 2A and 2B) in the first part of the research, and then to verify them in the second part for the original Toce River physical model (Figs. 2C and 2D). After the identification step, we chose the Full Momentum model as an appropriate mathematical representation of unsteady water flow in a built-up area. We also found the optimal modeling parameters, for instance, 1 cm is the proper mesh resolution, and the Manning value equal to 10 m^−1/3^⋅s is the best to represent building blocks in the flow field. Based on this, we prepared the models for the original DEM, to verify the modeling techniques.

**Figure 10:**
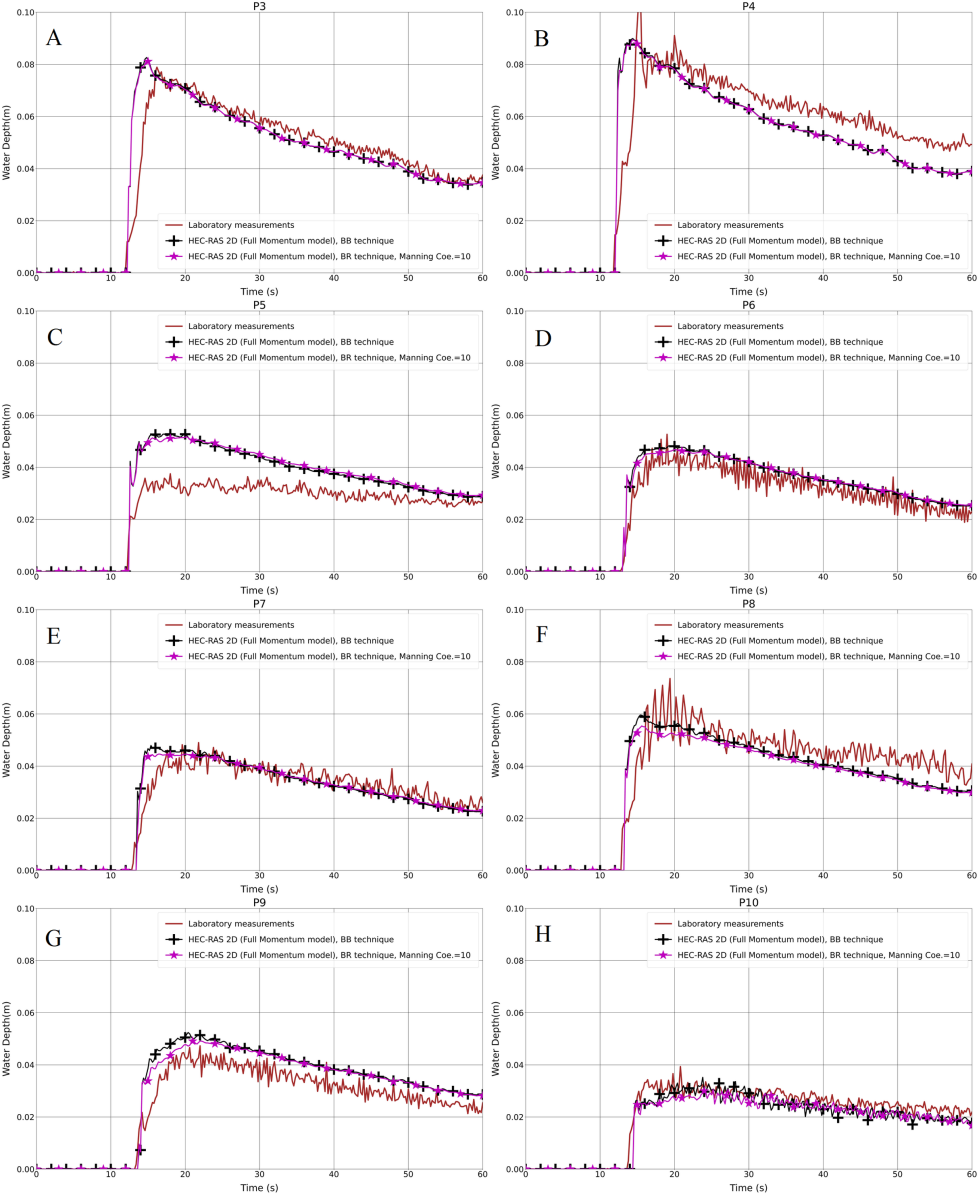
(A–H) Analysis of the numerical simulation using different building representation techniques for the original DEM with the aligned building layout (2aBB and 2a-BR).

**Figure 11:**
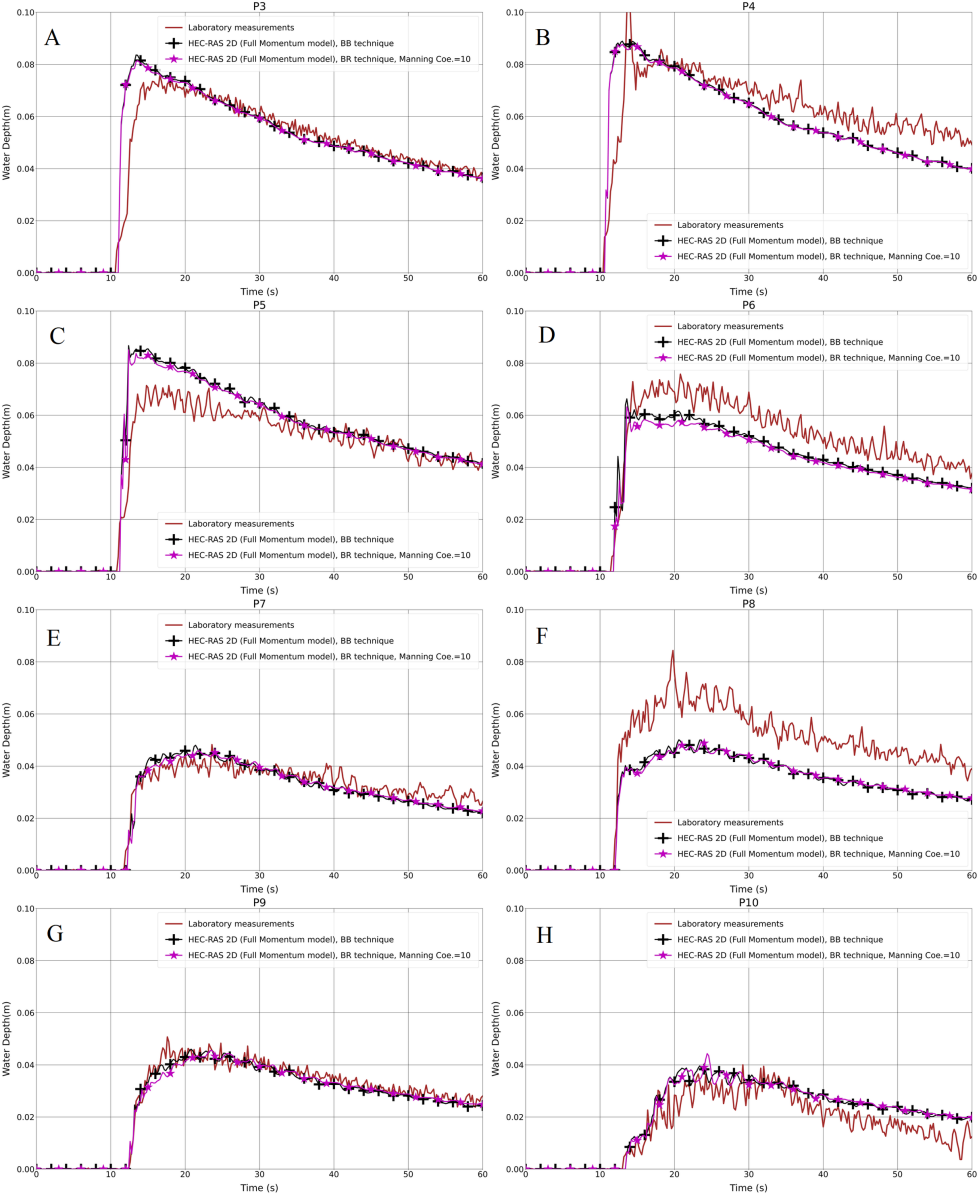
(A–H) Analysis of the numerical simulation using different building representation techniques for the original DEM with the staggered building layout (2b-BB and BR).

The investigated building setups in the second step of the work were just the same as previously. Figures 10A–10H and 11A–11H show the results for both setups and for the BB and BR techniques. With regard to the Full Momentum model in the BB technique for a 1 cm grid resolution, generally it fits well with the laboratory measurements in both building layouts. However, at P5 in the aligned layout (Fig. 10C), the water depth is overestimated and at P8 in the staggered layout (Fig. 11F), the water depth is underestimated. The underestimation of the experimental data observed in P8 was already observed by Costabile et al. (2020a) and previously also observed by other authors (Kim et al., 2014; Soares-Frazão et al., 2008).

Concerning the statistical analysis for water depth outcomes, the RMSE and PPMCC results for both techniques and in both layouts showed good consistency (Tables 6 and 7), being very close to each other. This verifies that both techniques have similar characteristics and the BR technique could be used instead of BB in the case of low-resolution DEMs.

**Table 6:** Calculated RMSE for both building layouts and different building representations (cm) (2a-BB, 2a-BR, 2b-BB and 2b-BR).

Building layout	Building representation technique	P3	P4	P5	P6	P7	P8	P9	P10
Aligned (2a)	BB	0.82	1.15	1.02	0.48	0.47	0.75	0.69	0.36
	BR	0.80	1.13	1.03	0.47	0.40	0.77	0.60	0.38
Staggered (2b)	BB	0.80	1.07	0.92	0.85	0.39	1.46	0.26	0.62
	BR	0.78	1.07	0.81	0.94	0.36	1.46	0.28	0.63

**Table 7:** Calculated PPMCC for both building layouts and different building representations (2a-BB, 2a-BR, 2b-BB and 2b-BR).

Building layout	Building representation technique	P3	P4	P5	P6	P7	P8	P9	P10
Aligned (2a)	BB	0.94	0.92	0.96	0.98	0.96	0.94	0.98	0.97
	BR	0.94	0.93	0.97	0.98	0.97	0.95	0.98	0.98
Staggered (2b)	BB	0.95	0.93	0.96	0.98	0.97	0.99	0.99	0.94
	BR	0.95	0.93	0.97	0.99	0.97	0.99	0.99	0.94

## Discussion

The capability of HEC-RAS 2-D to cope with the propagation of flood waves in built-up areas using the Toce River physical model has been assessed. Both available Full Momentum and Diffusion Wave hydrodynamic models were applied. Different building representation techniques in different building configurations and mesh resolutions were investigated. In light of the obtained outcomes, which were shown in the results section, the results are discussed in this section, as follows:

### Shallow water equations (SWEs) vs the diffusion wave model

Dottori & Todini (2013) observed that the water depths computed by the Diffusion Wave model inside the urban district are unfluctuating compared to the observed values. Costabile, Costanzo & Macchione (2017) concluded that the Diffusion Wave model is questionable, especially in the urban zone because of the poor prediction of the events that might be simulated in the vicinity of the buildings. Prestininzi (2008) stated that the parabolic approximation, even if it fails to reproduce some local phenomena such as high frequency oscillation, bores and run-up, is capable of simulating the propagation of such an impulsive wave over complex topography. From our results, we observed that generally, water depth gets underestimated at all probe points in the staggered layout using the Diffusion Wave model. In fact, the staggered layout is most similar to a typical the urban layout. As a result, we can conclude that using the Diffusion Wave model will produce inaccurate results. Finally, our numerical results are consistent with the previously mentioned results and conclusions that the Diffusion Wave model is not the proper model for urban flood simulation.

### Different building representation techniques

As shown previously in Figs. 6A–6H and 7A–7H, both the test cases (Full Momentum model and Diffusion Wave model) using the BB technique and the measurements were compared to the solution presented by Szydłowski (2005), who applied the Full Momentum hydrodynamic model and the BH technique to model the built-up area. Generally, there is a good correlation between the Full Momentum model applied in HEC-RAS 2-D and the numerical calculation by Szydłowski (2005). Both are a good fit with the laboratory measurements in both scenarios. In fact, both the BB and BH techniques work in approximately the same way to prevent water storage and water flow over the building units.

Lately, one of the most popular techniques/models to model urban flooding is Building Porosity (BP). Soares-Frazão et al. (2008) investigated the application of modified shallow-water equations with porosity in the frame of shock-capturing, Godunov-type algorithms (Godunov, 1959). Their main conclusions are that the porosity model is capable of reproducing the mean characteristics of the flow inside and around the urban zone, and the computational costs are much smaller than those of the classical shallow-water equations solved on a refined mesh. We found that the numerical results in both studies give an earlier wave arrival time, which is due to the defined upstream boundary condition. When compared to the porosity model (SPR) in their study, the BR techniques give better agreement with the experimental water depth measurements at probe point 5 in the staggered layout.

Jeong, Yoon & Cho (2012) presented a two-dimensional unstructured finite volume model based on SWEs and a well-balanced HLLC scheme. Their main conclusion regarding the Toce river physical case is that the water depth was classified into a rapid reduction segment and a slow reduction segment when the flood wave propagated from the front part of the urban areas to their rear part, and the initial arrival time in front of the urban areas appeared to have been reduced as the inflow volume increased. They observed relatively high-water depth zones and lag phenomena in front of the urban area, which was also noticed in our study. The cause of this observation is related to the obstacle effects of the building units. A porosity-based computational model for the SWEs was proposed by Ferrari et al. (2019), who adopted an isotropic storage porosity parameter and anisotropic friction. In the presence of low-friction regimes, the results produced by the proposed anisotropic scheme are similar to a high-resolution resolved building model, although the computational times are significantly shortened. When we compare the anisotropic porosity (AP) with the BR technique in our study, we can observe similar results in water depth measurements. The trends confirm that both models results agree with experimental measurements of the reflection of the flood wave against the building blocks and the consequent water level rise. Costabile et al. (2020a) observed that the hydraulic behavior of the flow is correctly reproduced only by the SWE model and, in a less accurate way, by the Porosity Model (PM) in which no simplifications were introduced in the momentum equations. In addition, they stated that due to the intrinsic limitations represented by the absence of inertial terms, the Diffusion Model (ZI) was unable to provide a satisfactory description of the local effects caused by the interactions between the flood flow and idealized buildings. In a visual comparison of the BR technique in our study and PM in their work, we found that the water depth results at probe points 3 and 4 in BR technique were more consistent with experimental measurements. While similar results have been observed also at the other gauges. The present findings of our numerical simulations using the BR technique in HEC-RAS 2-D have significant implications for modeling urban flooding in an easier and more simplified way compared to the other models. In spite of it is a simplified method, but the BR technique gives a similar quality of results as the most complex model like the porosity model.

Previously, the original DEM of the Toce River (Figs. 2C and 2D) was studied by Li et al. (2019) who studied the original terrain using the TELEMAC-2-D model. Our numerical results are consistent with the mentioned work. The authors also observed discrepancies at P5 in the aligned layout and at P8 in the staggered layout. There are a few explanations regarding these discrepancies: Li et al. (2019) stated that it may be an effect of the variation in water level influenced by the water jump and micro-topography. Szydłowski (2006) suggested that it may be because of an error in the measurement which could not be verified at this stage. Despite the discrepancies at these two points, we believe that our results compare well with the laboratory measurements. The outcomes of the BR technique have a similar trend to the BB technique results. We believe that this result emphasizes the validity of our models. Based on this, we could state that the BR technique is a good technique to represent building units in simulations of unsteady water flow using HEC-RAS 2-D.

**Figure 12:**
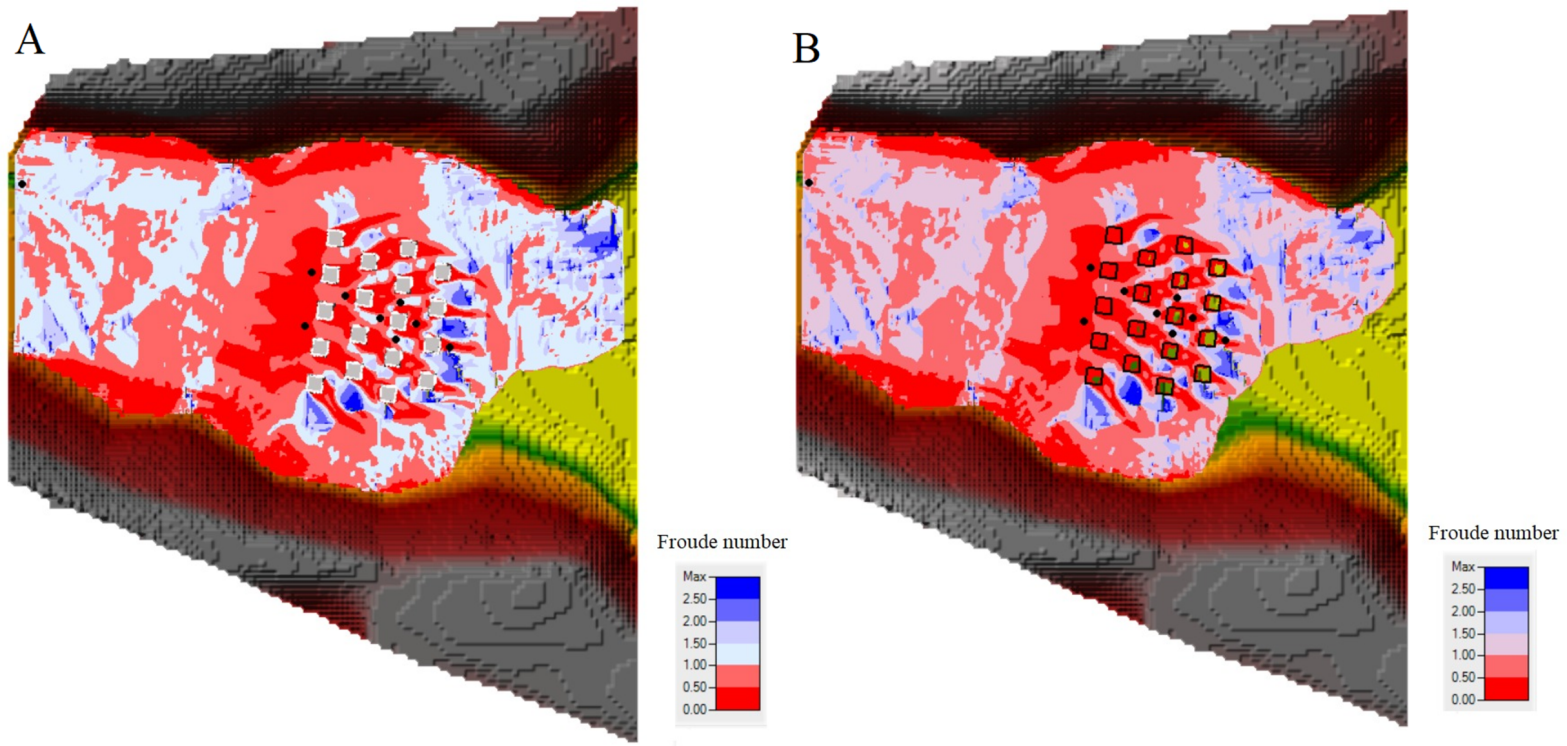
Froude number after 15 s: (A) BB technique with the staggered layout. (B) BR technique with the staggered layout.

**Figure 13:**
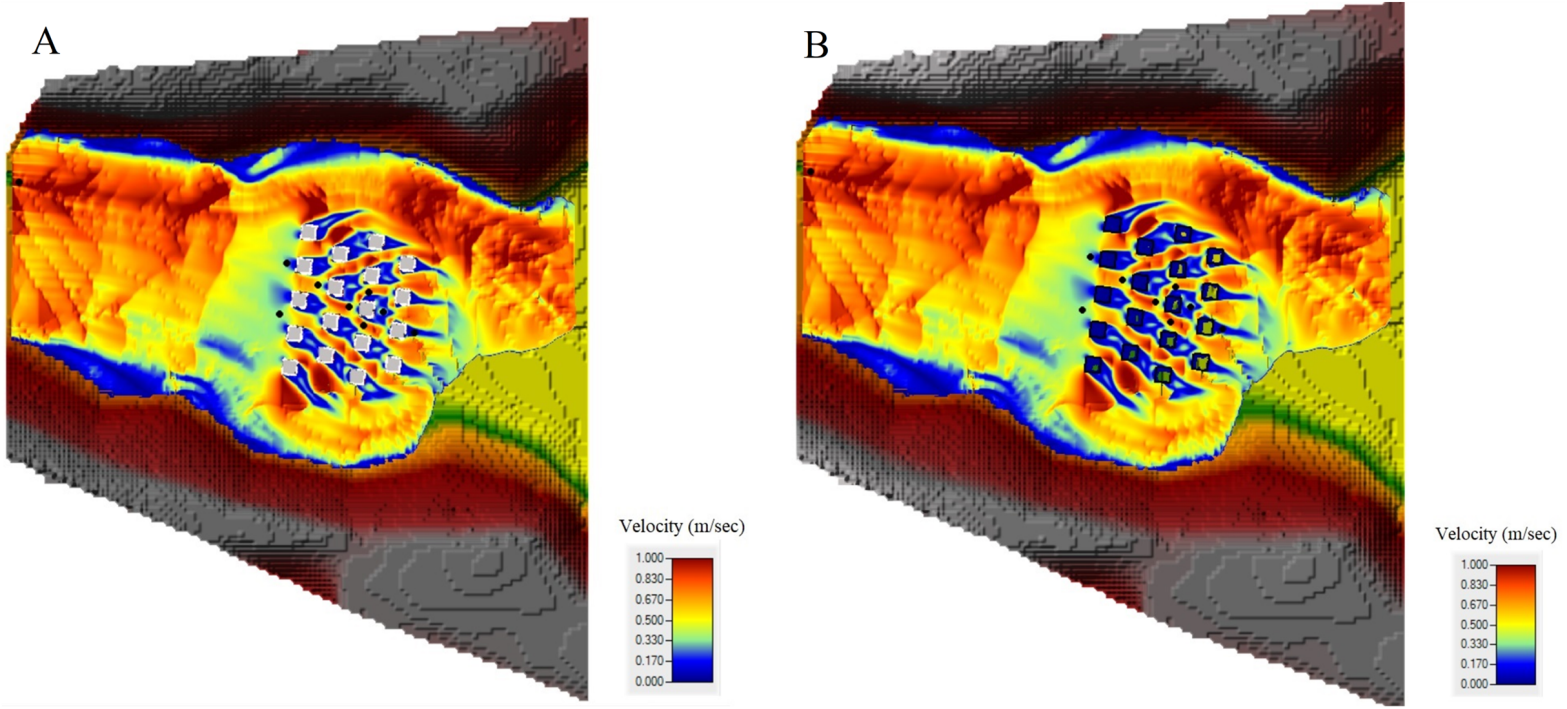
Velocity (m/s) after 15 s: (A) BB technique with the staggered layout. (B) BR technique with the staggered layout.

**Figure 14:**
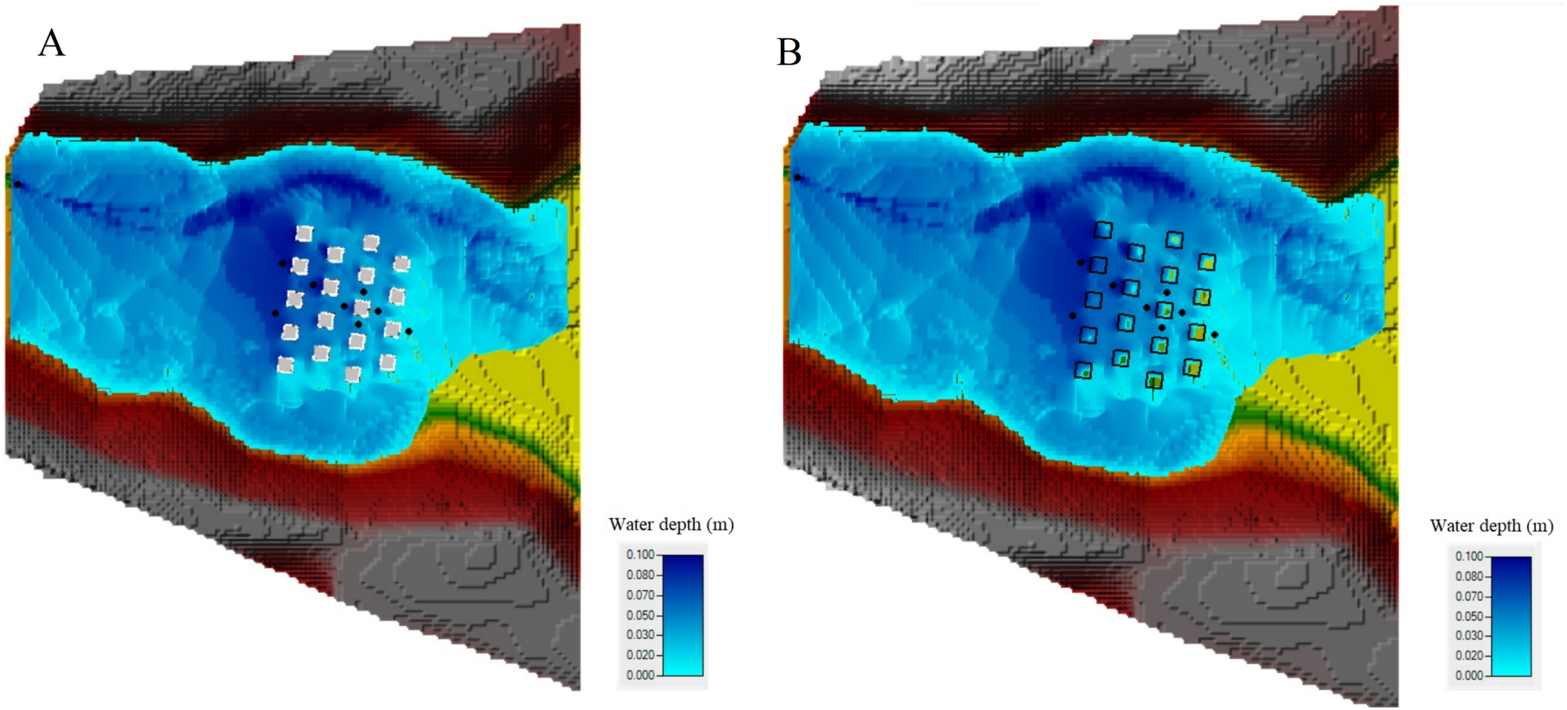
Water depth (m) after 15 s: (A) BB technique with the staggered layout. (B) BR technique with the staggered layout.

Additionally, in order to compare the BB and BR techniques, the flow structure in a built-up area was investigated by mapping the Froude number (Fr) (Figs. 12A and 12B), velocity field (Figs. 13A and 13B) and water depth maps (Figs. 14A and 14B) in the HEC-RAS 2-D RAS Mapper tool (the Froude number is defined here as the local velocity divided by the square root of the gravitation constant and depth). The maps were prepared only for the staggered layout, this scenario being more realistic, and were drawn up exactly after 15 s for the two building representation techniques (BB and BR). It can be seen that subcritical (Fr < 1) and supercritical (Fr > 1) flow areas, as well as regions of transcritical flow, are located at the same places. As it is shown in Figs. 12A and 12B, cases have been identified where different values of the Froude number occur: one case taking place at the upstream, where the Froude number is more than 1, and then just before the flow waves reach the buildings, the flow regime changed to subcritical, where the Froude number is less than 1. As a result of flow resistance, a hydraulic jump was formed in front of the built-up area. The jump was not a stationary phenomenon and it migrated upstream during the experiments. Under both building representation (BB and BR) techniques, the flow regimes around the building area are the same, particularly the interactions of subcritical and supercritical flows. Behind the buildings and in a forward flow direction, there is subcritical flow, whereas in the center of the buildings and in an onward flow direction, there are subcritical and supercritical flows around the buildings. Furthermore, HEC-RAS 2-D provides good performance during the transition from subcritical to supercritical flows.

Moreover, velocity maps were also prepared in the same way as the Froude number maps. Figure 13A shows that in the BB technique, the flow structure around the building is variable due to the buildings working as a barrier against the flow. While in the BR technique, the flow structure around the building is not that variable (Fig. 13B). Additionally, in the BR technique, a low-velocity value appeared inside the building units, the reason for which is because of the high Manning coefficient assigned to these specific polygons inside the 2-D flow area in HEC-RAS 2-D.

In order to better understand what happens in both situations, maps of the extent of the flooded area and the water level were prepared for both techniques. Figures 14A and 14B display the water extent and the color indicates the water depth level. There, the water level values vary between 0.01 and 0.1 m. Generally speaking, it can be noticed that the water depth levels computed using both techniques inside the urban district are very similar. Focusing on the water depth maps (Figs. 14A and 14B) for both techniques, BB and BR, it appears that the hydraulic jump that takes shape just ahead of the building blocks is correctly reproduced by both techniques. Generally, after 15 s of simulation, the total inundated extent in both techniques is the same. Finally, these techniques are used on scale models in HEC-RAS 2-D. Extreme caution should be taken when applying these approaches to full-scale models due to the scaling issues of various hydrodynamic variables.

### The impact of the building layout

Generally, the staggered layout exhibited more resistance to flood propagation than the aligned layout, thus inducing high flow velocity in the built-up area. The peak value of the water depth and the time to peak also showed variations among the mesh sizes. For instance, at the gauge points P3, P4 and P5, which were hit by the flow straightaway, the calculated peak water depth values are higher than the measured values compared to other gauge points, as well as at different mesh resolutions. With regard to the water depth, the tendency was not the same because the second and the fourth rows of buildings in the staggered layout worked as a barrier to the flow, consequently increasing the water depth and reducing the velocity upstream of the building. The mesh resolution has no significant impact on the predicted time of the water depth peak being ahead of the measurements. However, there is a tiny improvement in the 1 cm resolution compared to the 2 and 5 cm grid resolutions. Further analysis revealed that decreasing the mesh size causes data oscillation in the numerical modeling, which affects the accuracy of the results.

### The impact of the Manning coefficient

The artificial increase of the Manning coefficient is only within the areas of the buildings in the BR method, not the Manning coefficient for the whole 2-D flow field. The increase in the Manning coefficient has a significant impact up to a certain value, but a further increase appears to have no significant effect on the outcomes. Thus, the Manning coefficient would be considered as an important parameter in numerical modeling with the BR technique. When the Manning coefficient value is greater than 1, the performance of the numerical model improves, which is of great practical importance in such kinds of models. As flood modeling processes and the mapping of results, especially in urban areas, require a lot of data, such as accurate terrain data, which nowadays are expensive, accordingly, the BR technique is worth applying using high Manning coefficients with regard to the resistance effect of buildings.

## Conclusions

In conclusion, urban features such as apartments, houses, business buildings, roads and man-made infrastructures obviously affect urban flooding. Both water depth and velocity are the most important parameters in mapping flood risk and calculating damage assessment. This paper has investigated two simplified building layouts: aligned and staggered, in two methods of building representations: BB and BR. The HEC-RAS 2-D model was used to analyze unsteady urban flooding by taking the classical Toce River experimental test case. Based on this, eight models were prepared, analyzed and discussed. Water depth at all probe points from all models was compared to the measured values from the laboratory. The following are the main conclusions drawn from the work:

- The HEC-RAS 2-D model is able to simulate unsteady urban flooding in two methods of building representations. As far as BR is concerned, the technique is a good representation of building units in numerical simulations using high Manning coefficients.
- HEC-RAS 2-D presents two types of computational approaches in order to model the flow field in the defined 2-D mesh: Full Momentum (Saint-Venant) equations and the Diffusion Wave model. In the Diffusion Wave method, we noticed that the water depth at all probe points was underestimated, except at P10 where at this point the curve fits the laboratory measurement curve. The reason for this is the Diffusion Wave model is devoid of inertia force representations and it could not accurately calculate the water swelling and flow dynamics for the rapidly varied and transcritical water flow.
- Regarding the BR method, the value of the Manning coefficient is the crucial parameter, due to the fact that it represents the volume of water passing into the building grids. A remarkable and, in fact, powerful aspect of BR is that predictions of velocity and even water depth are very sensitive to the value of the Manning coefficient used for developed building units in the range of 1 or 10 m^−1/3^⋅s considered in the study. It is a good option when detailed building geometry data or DEMs are not available, and it can be used with any kind of computational mesh resolution.
- Only, with the probe points located at the back of the building blocks did the numerical results using the BR technique with the Manning coefficient equal to 0.1 m^−1/3^⋅s show a close correlation to the numerical simulations using higher Manning coefficients and the observed measurements. This is due to there being no strong flow structure because the buildings work as a barrier.
- The BR compared to the BB is the easiest technique to implement and is capable of relatively fast execution, but the BR does not provide the same precision as the BB technique, especially with respect to the velocity prediction.
- A sensitivity analysis of numerical models with 1, 2 and 5 cm mesh resolutions in the BB method was undertaken in order to show the sensitivity of different resolutions. Water depth results showed that the 1 cm mesh resolution fits relatively well with the laboratory measurements and other numerical models. The statistical analysis indicators RMSE and PPMCC were found to verify the accuracy of 1 cm compared to 2 and 5 cm mesh resolutions.

For comprehensive predictive modeling, including the accurate prediction of localized depths and velocities, more comprehensive urban flood validation datasets are required. Last but not least, these techniques are used on scale models in HEC-RAS 2-D, and their applicability to real-world case studies should be investigated. In particular, the building layout, roughness and flow hydrograph is different in real urban areas. The issue of the validity of the parameters in HEC-RAS 2-D at a larger scale is the subject of future research.

## Supplemental Information

10.7717/peerj.11667/supp-1 Supplemental Information 1 Input files to HEC-RAS modeling software. The raw input data for HEC-RAS modeling was obtained from G. Testa, D. Zuccala, F. Alcrudo, J. Mulet and S. Soares-Frazao, “Flash flood flow experiment in a simplified urban district” Journal of Hydraulic research, 2007, VOL 45 Extra Issue, 37-44 DOI: 10.1080/00221686.2007.9521831.

10.7717/peerj.11667/supp-2 Supplemental Information 2 The output of numerical simulation in HEC-RAS 2-D for the modified DEM using Building Resistance (BR) technique in the aligned configuration. The raw output data show the results of numerical simulations using different techniques, different models, different mesh resolutions, different Manning value, and different configuration layout.

10.7717/peerj.11667/supp-3 Supplemental Information 3 The output of numerical simulation in HEC-RAS 2-D for the modified DEM using Building Resistance (BR) technique in the staggered configuration.

10.7717/peerj.11667/supp-4 Supplemental Information 4 The output of numerical simulation in HEC-RAS 2-D for the modified DEM using Building Block (BB) technique in the aligned configuration.

10.7717/peerj.11667/supp-5 Supplemental Information 5 The output of numerical simulation in HEC-RAS 2-D for the modified DEM using Building Block (BB) technique in the staggered configuration.

10.7717/peerj.11667/supp-6 Supplemental Information 6 The output of numerical simulation in HEC-RAS 2-D for the original DEM in the aligned configuration.

10.7717/peerj.11667/supp-7 Supplemental Information 7 The output of numerical simulation in HEC-RAS 2-D for the original DEM in the staggered configuration.

## Additional Information and Declarations

### Data Availability

The following information was supplied regarding data availability: The raw inputs and outputs are available in the Supplemental Files. The experimental (laboratory) data is originally published by Testa et al. (2007) and is available online at DOI 10.1080/00221686.2007.9521831.
